# National Vaccination Coverage Among Adolescents Aged 13–17 Years — National Immunization Survey-Teen, United States, 2021

**DOI:** 10.15585/mmwr.mm7135a1

**Published:** 2022-09-02

**Authors:** Cassandra Pingali, David Yankey, Laurie D. Elam-Evans, Lauri E. Markowitz, Madeleine R. Valier, Benjamin Fredua, Samuel J. Crowe, Shannon Stokley, James A. Singleton

**Affiliations:** ^1^Immunization Services Division, National Center for Immunization and Respiratory Diseases, CDC; ^2^Division of Viral Diseases, National Center for Immunization and Respiratory Diseases, CDC; ^3^Leidos Health, Inc., Atlanta, Georgia; ^4^Division of Bacterial Diseases, National Center for Immunization and Respiratory Diseases, CDC.

CDC’s Advisory Committee on Immunization Practices (ACIP) recommends routine vaccination of persons aged 11–12 years with tetanus, diphtheria, and acellular pertussis vaccine (Tdap), human papillomavirus (HPV) vaccine, and quadrivalent meningococcal conjugate vaccine (MenACWY). A second (booster) dose of MenACWY is recommended at age 16 years. On the basis of shared clinical decision-making, adolescents aged 16–23 years may receive a serogroup B meningococcal vaccine (MenB) series. Catch-up vaccination is recommended for hepatitis A vaccine (HepA); hepatitis B vaccine (HepB); measles, mumps, and rubella vaccine (MMR); and varicella vaccine (VAR) for adolescents whose childhood vaccinations are not up to date (*1*). Although COVID-19 vaccination and influenza vaccination coverage estimates are not presented in this report, vaccination with a COVID-19 vaccine and annual influenza vaccination are also recommended by ACIP for adolescents* (*2*). To estimate vaccination coverage, CDC analyzed data for 18,002 adolescents aged 13–17 years from the 2021 National Immunization Survey-Teen (NIS-Teen).^†^ Coverage with ≥1 dose of Tdap^§^ (89.6%) and ≥1 dose of MenACWY^¶^ (89.0%) remained high and stable compared with the previous year. Increases in coverage with the following vaccines occurred from 2020 to 2021: ≥1 dose of HPV** vaccine (from 75.1% to 76.9%); adolescents who were up to date with HPV vaccination (HPV UTD)^††^ (from 58.6% to 61.7%); and ≥2 MenACWY doses among adolescents aged 17 years (from 54.4% to 60.0%). Coverage with MenACWY, HPV vaccine, and ≥2 HepA doses was lower among adolescents living in nonmetropolitan statistical areas (non-MSAs)^§§^ than among those living in MSA principal cities. The potential impact of the COVID-19 pandemic was assessed by comparing vaccination coverage by age and birth year before and during the COVID-19 pandemic. Coverage with ≥1 MenACWY dose by age 13 years was 5.1 percentage points lower among adolescents who reached age 13 years during the pandemic (2021) compared with those who reached age 13 in 2019. Coverage with ≥1 Tdap dose by age 12 years was 4.1 percentage points lower among children who reached age 12 years during the pandemic (2020) compared with those who reached age 12 before the pandemic. Coverage with ≥1 HPV vaccine dose by ages 12 and 13 years among children and adolescents who reached age 12 or 13 during the pandemic did not differ from coverage before the pandemic. Many children and adolescents might have missed routine medical care and recommended vaccinations during the COVID-19 pandemic. Review of patient vaccination records is important for providers to ensure that children and adolescents are up to date with all recommended vaccinations.

NIS-Teen is an annual random-digit–dialed telephone survey^¶¶^ that estimates vaccination coverage among adolescents aged 13–17 years in the 50 states, the District of Columbia, selected local areas, and some U.S. territories.*** Parents and guardians of age-eligible adolescents are interviewed about household sociodemographic characteristics and are asked for permission to contact the adolescent’s vaccination providers. Immunization history questionnaires are mailed to vaccination providers with the permission of the parent or guardian to obtain the adolescent’s vaccination record. Vaccination coverage estimates are based on provider-reported vaccination histories and include any vaccines administered before the 2021 NIS-Teen interview date. This report presents vaccination coverage estimates for 18,002 adolescents aged 13–17 years.^†††^ The overall Council of American Survey Research Organizations response rate^§§§^ was 21.0%; 41.2% of adolescents with completed interviews had adequate provider data. Data were weighted and analyzed to account for the complex survey design. T-tests were used to compare differences in vaccination coverage by survey year (2021 versus 2020) and among sociodemographic groups^¶¶¶^; differences with p<0.05 were considered statistically significant. The cumulative percentage of adolescents vaccinated by single year of age milestones was assessed using Kaplan-Meier estimates to account for censoring of vaccination status at ages ≥14 years, stratified by annual birth cohort (2002–2008). To assess potential COVID-19 pandemic effects for ≥1 HPV vaccine, ≥1 MenACWY, and ≥1 Tdap dose, vaccination coverage by age 12 years was compared for children born in 2008 (i.e., those who reached age 12 years in 2020, during the pandemic) to those born in 2007 (i.e., those who reached age 12 years in 2019, before the pandemic); vaccination coverage by age 13 years was compared for adolescents born in 2007 and 2008 (those who reached age 13 years in 2020 and 2021, respectively) to those born in 2006 (those who reached age 13 years in 2019). Analyses were conducted using SAS-callable SUDAAN (version 11; RTI International). This activity was reviewed by CDC and was conducted consistent with applicable federal law and CDC policy.****

## National Vaccination Coverage

**FIGURE 1:**
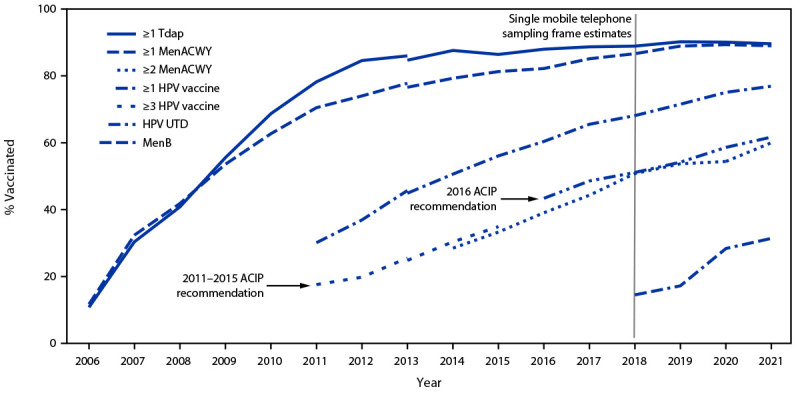
Estimated vaccination coverage with selected vaccines and doses*^,^^†^ among adolescents aged 13–17 years, by survey year — National Immunization Survey-Teen,^§^^,^^¶^ United States, 2006–2021 **Abbreviations:** ACIP = Advisory Committee on Immunization Practices; APD = adequate provider data definition; HPV = human papillomavirus; HPV UTD = up to date with HPV vaccination; MenACWY = quadrivalent meningococcal conjugate vaccine; MenB = serogroup B meningococcal vaccine; Tdap = tetanus, diphtheria, and acellular pertussis vaccine. * ≥1 dose Tdap at age ≥10 years; ≥1 dose MenACWY or meningococcal-unknown type vaccine; ≥2 doses MenACWY or meningococcal-unknown type vaccine among adolescents aged 17 years at time of interview. Does not include adolescents who received their first and only dose of MenACWY at age ≥16 years; HPV vaccine includes 9-valent, quadrivalent, or bivalent HPV vaccine. The routine ACIP recommendation for HPV vaccination was made for females in 2006 and for males in 2011. Because HPV vaccination was first recommended for males in 2011, coverage for all adolescents was not measured before that year; HPV UTD includes those with ≥3 doses, and those with 2 doses when the first HPV vaccine dose was initiated at age <15 years and at least 5 months minus 4 days elapsed between the first and second dose. ^†^ ACIP revised the recommended HPV vaccination schedule in late 2016. The schedule changed from a 3-dose to a 2-dose series with appropriate spacing between receipt of the first and second dose for immunocompetent adolescents initiating the series at age <15 years. Three doses are still recommended for persons initiating the series at age ≥15 years. Because of the change in definition, the graph includes estimates for ≥3 doses of HPV vaccine during 2011–2015 and the HPV UTD estimate during 2016–2021. Because HPV vaccination was first recommended for males in 2011, coverage for all adolescents was not measured before that year. ^§^ NIS-Teen implemented a revised APD in 2014 and retrospectively applied the revised APD to 2013 data. Estimates using different APDs might not be directly comparable. ^¶^ NIS-Teen moved to a single-sample frame in 2018.

In 2021, 89.6% of adolescents aged 13–17 years had received ≥1 Tdap dose and 89.0% had received ≥1 MenACWY dose (Figure 1) (Table). Coverage with ≥1 HPV vaccine dose in 2021 was 76.9%, an increase of 1.8 percentage points from 2020; 61.7% were HPV UTD, an increase of 3.1 percentage points. Among those aged 17 years, coverage with ≥2 MenACWY doses was 60.0%, an increase of 5.6 percentage points from 2020; coverage with ≥1 MenB dose was 31.4%. Coverage with ≥2 HepA doses was 85.0%, an increase of 2.9 percentage points from 2020. Coverage remained >90% for ≥2 doses of MMR, ≥3 doses of HepB, and both VAR dose among adolescents without a history of varicella disease.^††††^

**TABLE:** Estimated vaccination coverage with selected vaccines and doses among adolescents aged 13–17 years,* by age at interview — National Immunization Survey-Teen, United States, 2021

Vaccine	Age at interview, yrs, % (95% CI)^†^					Total % (95% CI)^†^	
	13	14	15	16	17	2021	2020
**Total no. of recipients**	**3,691**	**3,789**	**3,681**	**3,548**	**3,293**	**18,002**	**20,163**
**Tdap^§^ ≥1 dose**	87.4 (85.2–89.4)	90.4 (88.2–92.2)	91.4 (89.6–92.9)^¶^	88.7 (85.8–91.1)	90.0 (87.5–92.1)	**89.6 (88.6–90.5)**	**90.1 (89.2–90.9)**
MenACWY**							
≥1 dose	85.6 (82.8–88.0)	89.4 (86.4–91.8)^¶^	90.3 (88.4–91.9)^¶^	88.4 (85.6–90.8)	91.3 (89.2–93.0)^¶^	**89.0 (87.9–90.0)**	**89.3 (88.4–90.2)**
≥2 doses^††^	NA	NA	NA	NA	60.0 (56.6–63.3)	**60.0 (56.6–63.3)^§§^**	**54.4 (51.2–57.5)**
**HPV vaccine^¶¶^**							
**All adolescents**							
≥1 dose	72.5 (69.5–75.2)	74.1 (70.7–77.3)	79.0 (75.9–81.8)^¶^	78.9 (75.7–81.8)^¶^	80.4 (77.7–82.8)^¶^	**76.9 (75.6–78.2)^§§^**	**75.1 (73.9–76.2)**
HPV UTD***	49.4 (46.0–52.8)	59.4 (55.8–62.9)^¶^	66.2 (62.7–69.6)^¶^	65.8 (62.3–69.2)^¶^	67.9 (64.8–70.9)^¶^	**61.7 (60.2–63.2)^§§^**	**58.6 (57.3–60.0)**
**Female**							
≥1 dose	73.7 (69.4–77.6)	75.6 (70.7–79.9)	82.4 (78.6–85.7)^¶^	79.2 (73.8–83.6)	82.3 (78.2–85.7)^¶^	**78.5 (76.6–80.4)**	**77.1 (75.4–78.7)**
HPV UTD	50.1 (45.3–54.9)	61.5 (56.3–66.4)^¶^	68.6 (63.6–73.1)^¶^	69.0 (63.7–73.8)^¶^	70.6 (65.9–74.9)^¶^	**63.8 (61.5–65.9)**	**61.4 (59.5–63.3)**
**Male**							
≥1 dose	71.2 (67.1–75.1)	72.7 (67.8–77.1)	76.0 (71.1–80.3)	78.7 (74.8–82.1)^¶^	78.6 (75.0–81.9)**	**75.4 (73.5–77.2)**	**73.1 (71.5–74.8)**
HPV UTD	48.7 (43.8–53.7)	57.5 (52.5–62.3)^¶^	64.2 (59.2–68.9)^¶^	62.5 (57.6–67.2)^¶^	65.5 (61.2–69.6)^¶^	**59.8 (57.6–61.8)^§§^**	**56.0 (54.1–57.8)**
**MenB ≥1 dose^†††^**	NA	NA	NA	NA	31.4 (28.2–34.8)^¶^	**31.4 (28.2–34.8)**	**28.4 (25.5–31.5)**
**MMR ≥2 doses**	93.5 (91.5–95.0)	92.7 (90.1–94.6)	91.9 (88.7–94.2)	91.8 (89.8–93.5)	91.3 (89.1–93.2)	**92.2 (91.2–93.2)**	**92.4 (91.6–93.2)**
**Hepatitis A vaccine ≥2 doses^§§§^**	88.8 (86.5–90.7)	86.0 (83.0–88.6)	85.5 (82.2–88.3)	84.4 (82.1–86.5)^¶^	79.7 (76.9–82.3)^¶^	**85.0 (83.8–86.1)^§§^**	**82.1 (81.1–83.1)**
**Hepatitis B vaccine ≥3 doses**	92.9 (90.8–94.5)	93.4 (91.7–94.8)	92.9 (90.5–94.8)	91.0 (88.2–93.2)	91.1 (88.6–93.0)	**92.3 (91.3–93.1)**	**92.6 (91.8–93.3)**
**Varicella history/Vaccine doses**							
No history, ≥1 dose	96.7 (95.3–97.6)	95.8 (94.2–97.0)	93.6 (90.1–95.9)	94.8 (93.1–96.1)	93.8 (91.5–95.4)^¶^	**94.9 (94.0–95.7)**	**95.6 (94.9–96.2)**
No history, ≥2 doses	93.3 (91.2–94.9)	91.4 (88.6–93.6)	90.6 (87.2–93.1)	91.9 (90.0–93.4)	90.6 (88.1–92.5)	**91.5 (90.5–92.5)**	**91.9 (91.0–92.7)**
History of varicella^¶¶¶^	5.5 (4.4–6.9)	8.0 (5.6–11.3)	6.5 (5.2–8.2)	7.8 (6.2–9.7)^¶^	8.9 (6.9–11.3)^¶^	**7.3 (6.5–8.2)**	**8.4 (7.6–9.2)**
History of varicella or received ≥2 doses vaccine	93.6 (91.7–95.1)	92.1 (89.4–94.1)	91.2 (88.1–93.6)	92.5 (90.8–93.9)	91.4 (89.2–93.2)	**92.2 (91.2–93.1)**	**92.6 (91.7–93.3)**

**Abbreviations:** HPV = human papillomavirus; HPV UTD = up to date with HPV vaccination; MenACWY = quadrivalent meningococcal conjugate vaccine; MenB = serogroup B meningococcal vaccine; MMR = measles, mumps, and rubella vaccine; NA = not applicable; NIS-Teen = National Immunization Survey-Teen; Tdap = tetanus, diphtheria, and acellular pertussis vaccine.

* Adolescents (18,002) surveyed in the 2021 NIS-Teen were born during January 2003–January 2009.

^†^ Estimates with 95% CIs >20 might not be reliable.

^§^ Includes percentages receiving Tdap at age ≥10 years.

^¶^ Statistically significant difference (p<0.05) in estimated vaccination coverage by age; reference group was adolescents aged 13 years.

** Includes percentages of adolescents receiving MenACWY or meningococcal-unknown type vaccine.

^††^ ≥2 doses of MenACWY or meningococcal-unknown type vaccine. Calculated only among adolescents aged 17 years at time of interview. Does not include adolescents who received 1 MenACWY dose at age ≥16 years.

**^§§^** Statistically significant difference (p<0.05) compared with 2020 NIS-Teen estimates.

^¶¶^ Includes 9-valent HPV, quadrivalent HPV, or bivalent HPV vaccine. For ≥1 HPV vaccine dose measure and HPV UTD measure, percentages reported were among females and males combined (18,002) and for females only (8,423) and males only (9,579).

*** Includes adolescents with ≥3 doses, and those with 2 doses when the first HPV vaccine dose was initiated at age <15 years and there were ≥5 months minus 4 days between the first and second dose. This update to the HPV vaccine recommendation occurred in December 2016. https://www.cdc.gov/vaccines/programs/iis/cdsi.html

^†††^ ≥1 dose of MenB; calculated only among adolescents aged 17 years at time of interview. Administered on the basis of individual clinical decision.

^§§§^ In July 2020, the Advisory Committee on Immunization Practices revised recommendations for hepatitis A vaccination to include catch-up vaccination for children and adolescents aged 2–18 years who have not previously received hepatitis A vaccine at any age. https://www.cdc.gov/vaccines/hcp/acip-recs/vacc-specific/hepa.html

^¶¶¶^ Determined by parent or guardian report or provider records.

## Vaccination Coverage by Selected Characteristics

Compared with adolescents living in MSA principal cities, coverage among those in non-MSAs was 9.0 percentage points lower for ≥1 HPV vaccine dose, 8.8 percentage points lower for HPV UTD, 3.0 percentage points lower for ≥1 MenACWY dose, and 6.9 percentage points lower for ≥2 HepA doses. Among adolescents aged 17 years, coverage with ≥2 MenACWY doses was 11.8 percentage points lower for those living in non-MSAs than for those in MSA principal cities. Disparities between non-MSAs and MSA principal cities were statistically significant for adolescents living at or above the poverty level, but not for those living below the poverty level^§§§§^ (Supplementary Table 1, https://stacks.cdc.gov/view/cdc/120475). Coverage also varied by jurisdiction (Supplementary Table 2, https://stacks.cdc.gov/view/cdc/120476), race and ethnicity,^¶¶¶¶^ and health insurance status.*****

## COVID-19 Pandemic Effects

**FIGURE 2:**
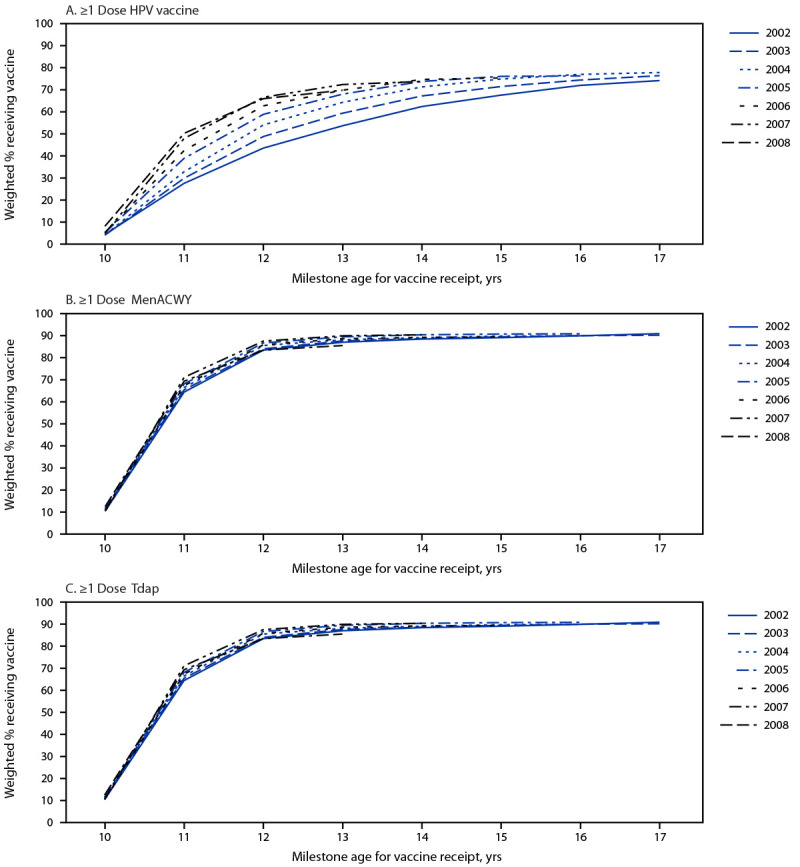
Coverage with ≥1 dose of human papillomavirus vaccine (A), ≥1 dose of quadrivalent meningococcal conjugate vaccine (B), and ≥1 dose of tetanus, diphtheria, and acellular pertussis vaccine (C), among adolescents in the 2002–2008 annual birth cohorts, by birth year and milestone age* — National Immunization Survey-Teen, United States, 2015–2021 **Abbreviations:** HPV = human papillomavirus; MenACWY = quadrivalent meningococcal conjugate vaccine; Tdap = tetanus, diphtheria, and acellular pertussis vaccine. * Milestone age is the age in years by which the cumulative percent of adolescents vaccinated was assessed and represents vaccination status up to but not including the birthday by which adolescents reached the indicated age.

Coverage with ≥1 HPV vaccine dose was higher at younger ages for adolescents born in more recent years (Figure 2).^†††††^ Coverage with ≥1 HPV vaccine dose by ages 12 and 13 years among children and adolescents who reached ages 12 and 13 years during the pandemic was similar to coverage among those who reached these milestone ages before the pandemic (Figure 2). Coverage with ≥1 MenACWY dose by age 13 years among adolescents who reached age 13 years during the pandemic was 5.1 percentage points lower (95% CI = −9.8 to −0.4) than among those who reached age 13 years before the pandemic. Coverage with ≥1 Tdap dose by age 12 years was 4.1 percent points lower (95% CI = −8.1 to −0.1) among children who reached age 12 years during the pandemic than among those who reached age 12 years before the pandemic. Tdap coverage by age 13 years among adolescents who reached age 13 years during the pandemic was not statistically different from coverage among those who reached age 13 years before the pandemic.

## Discussion

In 2021, coverage with ≥1 HPV vaccine dose, HPV UTD, and ≥2 HepA doses continued to increase among adolescents aged 13–17 years. Coverage with ≥1 Tdap dose, ≥1 MenACWY dose, ≥2 MMR doses, ≥3 HepB doses, and both doses of VAR among adolescents without a history of varicella disease remained high and stable. Coverage with ≥2 MenACWY doses among adolescents aged 17 years was higher in 2021 than in 2020.

Despite overall progress in vaccination coverage among adolescents, coverage disparities remain, particularly by MSA status. Coverage with MenACWY, HPV vaccine, and ≥2 HepA doses was lower among adolescents living in non-MSAs than among adolescents living in MSA principal cities. These geographic disparities were statistically significant only among adolescents living at or above poverty level. Access to the Vaccines for Children (VFC) program^§§§§§^ might contribute to higher vaccination coverage and lack of a geographic disparity for adolescents living below the poverty level among those in rural and urban areas. During 2016–2017, adolescents in rural areas were less likely than were those in urban areas to have had an age 11–12-year well-child visit (*3*), which might result in fewer opportunities to receive a vaccination and fewer opportunities to receive a recommendation for vaccination from a provider. However, differences might also stem from vaccine attitudes and beliefs because coverage was lower among those with incomes above poverty level. Confidence in vaccines has been lower in rural areas than in urban areas for both routine and COVID-19 vaccines (*4*,*5*).

Decreases in coverage with ≥1 MenACWY dose by age 13 years and ≥1 Tdap dose by age 12 years for children and adolescents born in 2008 suggest that disruptions to medical care during the COVID-19 pandemic resulted in lower coverage for these vaccines. Tdap coverage by age 13 years for adolescents born in 2008 was lower than coverage for those born in 2006, but the difference was not statistically significant. Data from eight health systems in the United States evaluating weekly vaccination rates and proportion of children up to date with all age-specific recommended vaccinations also indicated lower coverage during than before the pandemic (*6*). Large decreases in routine vaccination rates were found for children and adolescents aged 11–13 years during March 15–May 16, 2020, and the proportion of adolescents up to date with vaccinations by age 13 years was 3 percentage points lower in September 2020 (56%) than in September 2019 (59%). As more children who were aged 11–12 years when the COVID-19 pandemic was declared age into the NIS-Teen survey sample, the full impact of the COVID-19 pandemic can be better examined.

The findings in this report are subject to at least three limitations. First, the household response rate was 21.0%; 41.4% of completed interviews included adequate provider data. Bias from low response rates might occur if survey participants differ from nonparticipants (*7*). Second, although estimates are adjusted for household and provider nonresponse and households without a telephone, bias in the estimates might remain. A recent survey error assessment indicated that NIS-Teen estimates might underestimate true coverage, with the largest underestimation for Tdap (−6.3 percentage points).^¶¶¶¶¶^ Little evidence exists for a change in accuracy of NIS-Teen estimates from 2020 to 2021.****** Finally, this report did not assess the possible impact of the COVID-19 pandemic on adolescent vaccination at ages >13 years. An additional analysis of NIS-Teen data indicated no differences in coverage for adolescents aged 14–17 years during the pandemic compared with coverage before the pandemic.^††††††^

Achieving and maintaining high vaccination coverage levels for adolescents will ensure they have protection from serious and sometimes life-threatening vaccine-preventable diseases. To help adolescents catch up on missed vaccinations, health care providers can identify those who have fallen behind on receiving recommended vaccinations and remind families to schedule an appointment. In addition, during every clinical encounter, including those for COVID-19 vaccination, providers can review patients’ vaccination histories and recommend vaccination if needed. Resources to help promote and discuss vaccination with parents and patients can be found at https://www.cdc.gov/vaccines/hcp/patient-ed/index.html.

**Summary**

**What is already known about this topic?**

Tetanus, diphtheria, and acellular pertussis vaccine (Tdap), meningococcal conjugate vaccine (MenACWY), and human papillomavirus (HPV) vaccine are routinely recommended for adolescents.

**What is added by this report?**

Among adolescents aged 13–17 years in 2021, HPV vaccination coverage (≥1 dose and HPV vaccine up to date) increased. Coverage with ≥1-dose Tdap and ≥1-dose MenACWY remained high. Among age-eligible adolescents, MenACWY booster dose coverage increased. Analyses of the potential COVID-19 pandemic effect among adolescents born in 2008 show a concerning decrease in ≥1 MenACWY and ≥1 Tdap dose coverage.

**What are the implications for public health?**

As more adolescents who were due for routine vaccinations during the pandemic age into the NIS-Teen sample, the full impact of the pandemic can be assessed. Providers should review vaccination records to ensure that adolescents are current with all recommended vaccinations.

## Footnotes

* Influenza vaccination is recommended for all persons aged ≥6 months. Influenza vaccination coverage estimates are available at https://www.cdc.gov/flu/fluvaxview/index.htm. COVID-19 vaccination has been recommended within the scope of the Emergency Use Authorization by ACIP for children and adolescents aged ≥12 years since May 12, 2021. Estimates of COVID-19 vaccination coverage are available at https://covid.cdc.gov/covid-data-tracker/#vaccination-demographics-trends and https://www.cdc.gov/vaccines/imz-managers/coverage/covidvaxview/interactive/children.html.

† Eligible adolescents were born January 2003–January 2009. Estimates in this report include those who might have received on-time or catch-up vaccinations.

§ Tetanus toxoid, reduced diphtheria toxoid, and acellular pertussis vaccine represents coverage with ≥1 Tdap dose at age ≥10 years.

¶ Meningococcal conjugate vaccine represents coverage with the quadrivalent meningococcal conjugate vaccine or meningococcal-unknown type vaccine.

** HPV vaccination coverage includes receipt of any HPV vaccine and does not distinguish between 9-valent, quadrivalent, or bivalent vaccines.

†† HPV UTD includes adolescents with ≥3 doses, and those with 2 doses when the first HPV vaccine dose was initiated at age <15 years and there was ≥5 months minus 4 days between the first and second dose (https://www.cdc.gov/vaccines/programs/iis/cdsi.html). This update to the HPV vaccine recommendation occurred in December 2016. Some adolescents might have received more than the 2 or 3 recommended HPV vaccine doses.

§§ MSA status was determined from household reported city and county of residence and was grouped into three categories: MSA principal city, MSA nonprincipal city, and non-MSA. MSA and MSA principal city were as defined by the U.S. Census Bureau (https://www.census.gov/programs-surveys/metro-micro.html). Non-MSAs include urban populations not located within an MSA and completely rural areas.

¶¶ Persons living in all identified households with a mobile telephone were eligible for interview. Sampling weights were adjusted for single frame (mobile telephone), nonresponse, and noncoverage. A description of NIS-Teen single-frame survey methodology and its effect on reported vaccination estimates is available at https://www.cdc.gov/vaccines/imz-managers/coverage/teenvaxview/pubs-presentations/dual-to-single-frame-teen.html.

*** Local areas that received federal immunization funds under Section 317 of the Public Health Service Act were sampled separately. Those areas included Chicago, Illinois; New York, New York; Philadelphia County, Pennsylvania; Bexar County, Texas; and Houston, Texas. Three territories were sampled separately in 2021: Guam, Puerto Rico, and U.S. Virgin Islands.

††† The 2021 NIS-Teen sample included 8,423 females and 9,579 males. Adolescents from Guam (225), Puerto Rico (350), and U.S. Virgin Islands (245) were excluded from the national estimates.

§§§ The Council of American Survey Research Organizations response rate is the product of three other rates: 1) the resolution rate (the proportion of telephone numbers that can be identified as either business or residence), 2) the screening rate (the proportion of qualified households that complete the screening process), and 3) the cooperation rate (the proportion of contacted eligible households for which a completed interview is obtained).

¶¶¶ NIS-Teen methodology for weighting and synthesizing provider-reported vaccination histories has been previously described. https://www.cdc.gov/vaccines/imz-managers/nis/downloads/NIS-TEEN-PUF20-DUG.pdf

**** 45 C.F.R. part 46.102(l)(2); 21 C.F.R. part 56; 42 U.S.C. Sect. 241(d); 5 U.S.C. Sect. 552a; 44 U.S.C. Sect. 3501 et seq.

†††† Hepatitis A, hepatitis B, varicella, and measles, mumps, and rubella vaccines are considered childhood vaccinations and are recommended for adolescents who are not up to date with these vaccinations. Except as noted, coverage estimates for ≥1 and ≥2 varicella vaccine doses were obtained among adolescents with no history of varicella disease.

§§§§ Adolescents were classified as being below the federal poverty level if their total family income was less than the level specified for the applicable family size and number of children and adolescents aged <18 years. All others were classified as at or above the poverty level (https://www.census.gov/data/tables/time-series/demo/income-poverty/historical-poverty-thresholds.html). Poverty status was unknown for 579 adolescents.

¶¶¶¶ Hispanic or Latino (Hispanic) adolescents had lower coverage with ≥2 MenACWY doses (−10.8 percentage points), and ≥2 MMR doses (−3.6 percentage points) than did non-Hispanic White (White) adolescents. Non-Hispanic Black or African American adolescents had higher coverage with ≥1 HPV vaccine dose (7.1 percentage points) and for proportion HPV UTD (5.0 percentage points) than did White adolescents. Non-Hispanic Asian and non-Hispanic American Indian or Alaska Native (AI/AN) adolescents had higher coverage with most vaccines compared with White adolescents. https://www.cdc.gov/vaccines/imz-managers/coverage/teenvaxview/pubs-presentations/NIS-teen-vac-coverage-estimates-2021-tables.html#table-01

***** Adolescents with any Medicaid insurance had higher coverage with ≥1 HPV vaccine dose (4.6 percentage points) compared with adolescents with private health insurance. Adolescents who were uninsured had lower coverage with ≥1 MenACWY dose, ≥1 HPV vaccine dose, and HPV UTD compared with adolescents with private health insurance. https://www.cdc.gov/vaccines/imz-managers/coverage/teenvaxview/pubs-presentations/NIS-teen-vac-coverage-estimates-2021-tables.html#table-02

††††† NIS-Teen data during 2016–2021 were combined, and Kaplan-Meier methods were used to calculate cumulative vaccination coverage estimates by age in days, stratified by annual birth cohort (2006 = 9,992; 2007 = 5,914; and 2008 = 1,735).

§§§§§ Children and adolescents aged ≤18 years who are Medicaid-eligible, uninsured, or AI/AN (as defined by the Indian Health Care Improvement Act) are eligible to receive vaccines from providers through VFC. Children categorized as underinsured because their health plans do not include coverage with recommended vaccinations are eligible to receive VFC vaccines if they are served by a rural health clinic or federally qualified health center or under an approved deputization agreement. https://www.cdc.gov/vaccines/programs/vfc/providers/eligibility.html

¶¶¶¶¶ An assessment of validity of the 2020 NIS-Teen estimates has been reported (https://www.cdc.gov/vaccines/imz-managers/nis/downloads/NIS-TEEN-PUF20-DUG.pdf). NIS-Teen vaccination coverage estimates tended to be slightly lower compared with actual values derived after adjusting for noncoverage, nonresponse, and vaccination underascertainment, reaching up to 6.3 percentage points too low for Tdap. This was primarily attributed to underascertainment of vaccinations by NIS provider record check. The validity of estimates did not change from 2019 to 2020.

******
https://www.cdc.gov/vaccines/imz-managers/coverage/teenvaxview/pubs-presentations/NIS-teen-vac-coverage-estimates-2021-tables.html#table-03

††††††
https://www.cdc.gov/vaccines/imz-managers/coverage/teenvaxview/pubs-presentations/NIS-teen-vac-coverage-estimates-2015-2021.html
